# Transient Heat Stress During Early Seed Development Primes Germination and Seedling Establishment in Rice

**DOI:** 10.3389/fpls.2018.01768

**Published:** 2018-12-05

**Authors:** Kevin Begcy, Jaspreet Sandhu, Harkamal Walia

**Affiliations:** Department of Agronomy and Horticulture, University of Nebraska-Lincoln, Lincoln, NE, United States

**Keywords:** heat stress, seed development, seed priming, ABA-GA crosstalk, endosperm

## Abstract

Rice yield is highly sensitive to increased temperature. Given the trend of increasing global temperatures, this sensitivity to higher temperatures poses a challenge for achieving global food security. Early seed development in rice is highly sensitive to unfavorable environmental conditions. Heat stress (HS) during this stage decreases seed size and fertility, thus reducing yield. Here, we explore the transgenerational phenotypic consequences of HS during early seed development on seed viability, germination, and establishment. To elucidate the impact of HS on the developmental events in post-zygotic rice seeds, we imposed moderate (35°C) and severe (39°C) HS treatments initiated 1 day after fertilization and maintained for 24, 48, or 72 h. The transient HS treatments altered the initiation of endosperm (ED) cellularization, seed size and/or the duration of spikelet ripening. Notably, seeds exposed to 24 and 48 h moderate HS exhibited higher germination rate compared to seeds derived from plants grown under control or severe HS. A short-term HS resulted in altered expression of Gibberellin (GA) and ABA biosynthesis genes during early seed development, and GA and ABA levels and starch content at maturity. The increased germination rate after 24 of moderate HS could be due to altered ABA sensitivity and/or increased starch level. Our findings on the impact of transient HS on hormone homeostasis provide an experimental framework to elucidate the underlying molecular and metabolic pathways.

## Introduction

Rice (*Oryza sativa* L.) is one of the most important crops for global food security. Together with maize and wheat, these three cereals provide more than 50% of the food calories for humans as well as livestock feed, especially in Asia, Africa, and Latin America. Rice is grown under a variety of environments with an estimated planted area of 161 million ha (Seck et al., 2012). Current global climate predictions are expected to negatively impact rice production. It is estimated that increased temperatures will decrease rice production by up to 25% (Wassmann et al., 2009; van Oort and Zwart, 2018). Two major factors causing decline in rice yield are: heat stress (HS) induced sterility and shortening of the growing season (Tao et al., 2008; Li et al., 2015; van Oort and Zwart, 2018).

A shorter growing season could be particularly detrimental during the reproductive development. Increased temperatures following fertilization negatively affect grain development (Folsom et al., 2014; Begcy and Walia, 2015; Chen et al., 2016; Begcy and Dresselhaus, 2018). Accelerated seed development and reduced seed size are the most significant consequences of HS during early seed development (Folsom et al., 2014). The process of grain filling, which involves nutrient accumulation in developing and maturing grains, is also sensitive to environmental conditions and has strong effects on final yield and quality (Yang and Zhang, 2006). Early seed development entails an initial free nuclear stage (syncytial) and is followed by cellularization of endosperm (ED) nuclei. The cellularized ED continues to undergo mitotic divisions. The rate and duration of nuclear divisions and subsequent mitotic cell divisions are important determinants of sink capacity during grain filling stage. Environmental perturbations during the initial phases likely affect later stages of seed development and consequently grain size (Li et al., 2018). Several regulatory and metabolic pathways have been associated with abiotic stress induced seed development (Borisjuk et al., 2004; Whittle et al., 2009; Folsom et al., 2014; Begcy and Walia, 2015; Hu et al., 2015; Chen et al., 2016). Most acute and sustained HS studies only report treatment effects on seed size and molecular network patterns for the generation in which the stress was applied (Grass and Burris, 1995; Suzuki et al., 2013; Folsom et al., 2014). The transgenerational impact of HS is much less characterized. HS during seed development significantly affects seed quality, dormancy, germination, and emergence as well as seedling establishment (Khan, 1976; Finkelstein et al., 2008; Brunel-Muguet et al., 2015). Increased temperatures had a strong negative effect on seed germination potential and resulted in reduced seed viability and poor germination (Fahad et al., 2017). Decreased germination and seed vigor due to HS have been associated with reduced thermostability of the plasma membrane as well as membrane fluidity (Saidi et al., 2010; Fahad et al., 2017), which delayed activation of Ca^2+^ signaling, kinases, and heat shock factors (Sangwan et al., 2002; Hofmann, 2009; Saidi et al., 2010).

Hormonal and other chemical treatments can improve seed germination, establishment, and seedling vigor through a process called seed priming. In rice, seed priming is used to achieve faster and more uniform emergence (Farooq et al., 2006; Hussain et al., 2017; Ruttanaruangboworn et al., 2017). Seed priming has agricultural applications and will likely gain more importance as farmers seek greater resilience in their production practices under shifting weather patterns. A better understanding of seed priming and its modes of activity would therefore be of considerable value. Two major phytohormones, ABA and Gibberellin (GA) play antagonistic role in response to stress and during normal plant development, especially during seed germination. Endogenous ABA and GA levels negatively and positively regulate seed germination, respectively (Koornneef et al., 1982; White et al., 2000). A Comparison of dormant and non-dormant rice cultivars showed that ABA and GA exhibit dynamic fluxes during early, late and middle stages of seed development (Liu et al., 2014). In addition, exposure to HS can cause increase in ABA levels, while decreasing GA biosynthesis (Toh et al., 2008). In this study, we aimed to elucidate the effect of transient HS during early seed development on germination and seedling vigor of seeds derived from control and stressed plants. Our data suggest that the rate of seed germination depends upon the duration and intensity of thermal stress to which maternal plants were exposed. To understand the physiological mechanisms underlying altered germination, we examined the ABA, GA and starch contents of mature seeds. We also analyzed the temperature induced changes in expression of genes involved in GA and ABA metabolism during early seed development.

## Materials and Methods

### Plant Material and Heat Stress Treatment

Rice seeds (*O. sativa* var. Kitaake) were germinated on filter paper moistened with autoclaved double-distilled water (ddH_2_O) under dark conditions. The germination was conducted in growth chambers (I-36VL; Percival Incubators) at a constant temperature of 28°C. After germination, seedlings were transplanted to field soil in 4′ square pots. Plants were grown in standing water under controlled conditions (27–30°C) in the greenhouse until approaching anthesis. Plants were transferred to growth chambers 1–2 days before panicle emergence with a 16 h-light/8 h-dark cycle (300 – 400 μmol photons m^-2^s^-1^) at 28°C for non-stressed (NS) control conditions and 35 and 39°C for moderate and severe HS, respectively, and a relative humidity of 75 to 80%.

Unfertilized ovules were observed in the morning and then closely observed before the fertilization event. Closed spikelets with anthers located outside were then marked as fertilized by the afternoon. Plants were kept in control conditions until 24 h after fertilization (HAF) to assure that the marked seeds underwent a normal fertilization process and embryo and ED development processes have initiated. Post-fertilization HS was imposed for 24, 48, or 72 h. Only a few spikelets were collected from each plant. After the HS treatment, plants were moved back to the greenhouse and grown under control conditions until maturity for final seed phenotypic characterization. For all measurements, control and HS seeds were obtained from at least 20 plants for each of the five replicates. In rice, seed ripening involves changes in hull coloration from pale green to yellow-brown. Yellow-brown colored seeds were scored as ripened. For all seed ripening measurements, at least 20 plants in each of the four replicates from NS and HS seeds were used. Ripening rate was measured by scoring ripening every 4 days after fertilization.

### Germination and Post-germination Assay

Surface-sterilized dehulled seeds were germinated on wet filter paper in dark. Seeds obtained from plants exposed to HS and NS control conditions during early seed development were used for germination test. Seeds that exhibited radicle and coleoptile emergence were scored as germinated. Germination rate was measured by scoring germination every 6 h after imbibition (HAI) for 3 days. Assays were repeated four times and 20 seeds per treatment were used for each experimental replicate. To further test whether the hormone signaling of HS seeds affected, we applied 5 μM ABA (2-*cis*-4-*trans*-abscisic acid, 98% synthetic, Sigma-Aldrich) and 1 μM GA (Gibberellic acid, 95%, Sigma-Aldrich) in water on germinating seeds. For post-germination assays, seedling growth was measured at 5, 8, and 10 days after imbibition (DAI).

### Histological Analysis

Rice plants were prepared as above and three independent experiments were performed to collect seeds for sectioning. The images of seed sections in Figure 3 are representative of our observations across the replicates. For each time point, 20 developing rice seeds were harvested at 24, 48, 72, and 96 HAF, then fixed in a solution constituted of 3.7% formaldehyde, 5% acetic acid, and 50% ethanol at 4°C overnight. Samples were dehydrated through a graded ethanol series and infiltrated with xylene. Seed samples were embedded in paraffin (Fisher Scientific, Hampton, NH, United States), sectioned at a thickness of 10 μm, and stained with 0.1% toluidine blue. Sections were observed and photographed using a bright-field microscope (Leica DM-2500).

### Total Starch Analysis

One hundred grams of mature control and HS dry rice grains were used for starch analysis. Freeze-dried rice grains were powdered in a tissue lyser (TissueLyser, Retsch, Qiagen) operating at 30 Hz for 60 s and passed through a 0.5 mm screen. Soluble sugars were removed by washing the samples in 80% (v/v) ethanol, in a boiling water bath for 30 min, then in 96% ethanol at room temperature for a final wash for 10 min. The supernatant was removed at the end of every step after centrifugation at 10,000 ×*g* for 5 min, and the pellet was dried at 40°C. The pellet was re-suspended using 2 M KOH and 1.2 M sodium acetate buffer (pH 3.8). A total of 0.2 mL of a mixture of amyloglucosidase (1630 U/mL) plus invertase (500 U/mL; from Megazyme assay kit, Cat. No. K-YBGL) was added immediately. The contents were mixed well, and the tubes were incubated at 40°C for 30 min according to the protocol described by the manufacturer (Megazyme, Ireland). Absorbance for each sample and D-Glucose standards were read at 510 nm against the blank.

### Hormone Quantification

Mature NS, moderate (35°C) and severe (39°C) HS seeds were imbibed in water for 8 h (Howell et al., 2009; Rajjou et al., 2012), and then hormones of three biological replicates were extracted using high-performance liquid chromatography-mass spectrometry as described by Schmitz et al. (2015). Briefly, frozen seed material was grounded into powder with a mortar and pestle in liquid nitrogen. For each replicate, 100 mg (fresh weight) of tissue were used. Then, 500 μL of extraction solvent, 2-propanol/H_2_O/concentrated HCl (2:1:0.002, vol/vol/vol), were added to each tube. Samples were transferred to a shaker at a speed of 100 r.p.m. for 30 min at 4°C. Next, 1 ml dichloromethane was added to each sample and shaked for 30 min at 4°C, following a 5 min centrifugation step at 13,000 ×*g*. After centrifugation, two phases were formed; plant debris were distributed between the two layers. Approximately 900 μL of the solvent was transferred from the lower phase using a Pasteur pipette into a screw-cap vial and the solvent mixture was concentrated using a nitrogen evaporator with nitrogen flow. All samples were re-dissolved in 0.1 ml methanol. Finally, 50 μL of each sample solution was injected into the reverse-phase C18 Gemini HPLC column for HPLC–ESI–MS/MS analysis. A standard curve was calculated using GA20 (OlChemIm Ltd., Cat. 122481) to quantify extracted hormone levels.

### Statistical Analysis

For gene expression analysis, RNAseq data from NS (28°C) and HS (35 and 39°C) developing rice seeds after 24 and 48 h of HS mapped to the rice ssp. japonica genome (version 7.0) and were mined for hormonal response to HS (Chen et al., 2016). Statistical analyses and heat maps of expression profiles of genes involved in biosynthesis and catabolism of ABA and GA were performed using the R software/environment version 3.4.4 using data from three or more independent experiments (*n* = 20 or more plants) as described by Begcy and Dresselhaus (2017). A false discovery rate (FDR) < 0.0001 was used. For all experiments, we performed a *t*-test analysis to test the differences between control and treated seeds. Differences in means were considered significant at *p*-value < 0.05.

## Results

### Transient Heat Stress During Early Seed Development Negatively Impacts Endosperm and Embryo Development

**FIGURE 1:**
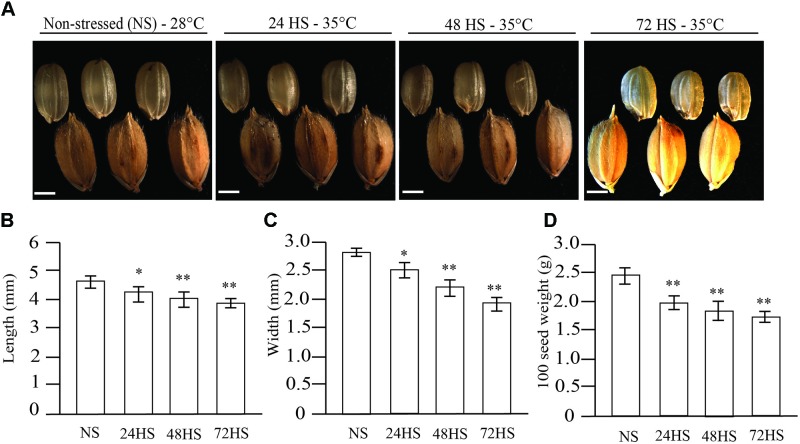
Morphometric analysis of mature rice seeds harvested from non-stressed (NS) plants (28°C) and plants exposed to moderate heat stress (HS; 35°C). **(A)** Comparison of mature rice seeds with and without husk between NS seeds (28°C) and moderate HS seeds at 35°C for 24, 48, or 72 h, imposed 24 h after fertilization. **(B)** Length; **(C)** Width; and **(D)** Weight. Length, width, and weight of dehulled seeds were significantly (^∗^*p*-value *a* < 0.001, ^∗∗^*p*-value < 0.0001, *n* = 100) reduced by HS. Bar = 1 mm.

**FIGURE 2:**
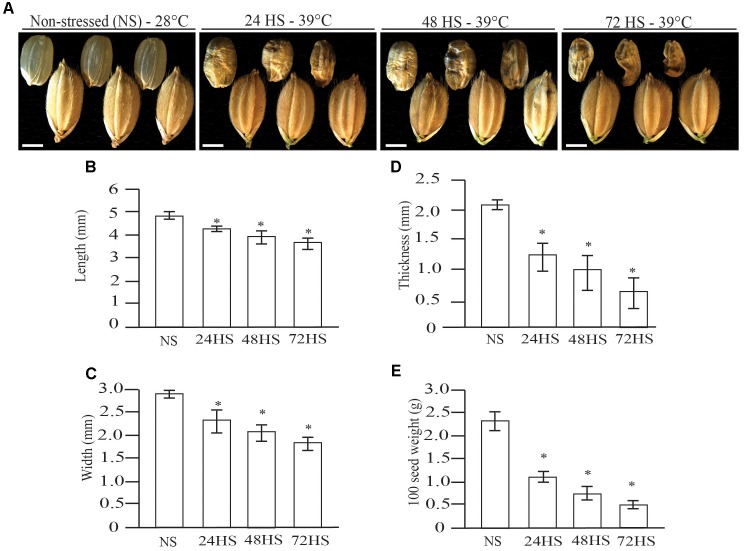
Seed malformation in mature rice seeds of plants NS (28°C) and plants exposed to 39°C HS. **(A)** Comparison of mature rice seeds with and without husk between NS seeds (28°C) and 39°C HS seeds for 24, 48, or 72 h, imposed 24 h after fertilization. **(B)** Length; **(C)** Width; **(D)** Thickness; and **(E)** Weight. Length, width, thickness, and weight of dehulled seeds were significantly (^∗^*p*-value < 0.0001, *n* = 100) reduced by a different HS duration. Bar = 1 mm.

To observe the effects of HS imposed during early ED development on mature rice seeds, we imposed a moderate (35°C) and a severe (39°C) HS for 24, 48, or 72 h initiated at 24 HAF. We observed that moderate HS (35°C) during early seed development significantly reduced the final seed size at maturity (Figure 1A). The reduced size of HS seeds at 35°C was a result of reduced seed length, width, and weight in mature seeds (Figures 1B–D). We also observed embryo malformation in seeds HS for 72 h at 35°C (Figure 1A). Embryo malformation was characterized by irregular shape and reduced size relative to control seeds. Seeds exposed to severe HS (39°C) during early seed development exhibited variable degree of malformation at maturity. The duration of 39°C HS directly impacted the severity of mature seed phenotypes (Figure 2A). The decrease in seed size after 24, 48, or 72 h at 39°C of HS was clearly reflected in reduced seed length, width, thickness, and weight at maturity (Figures 2B–E). We observed a significant decrease in seed weight (Figure 2D), indicating that even a short duration severe HS during early seed development results in collapse of ED. Seed viability of 39°C HS seeds (Figure 2A) was significantly reduced.

### Developmental Transitions During Early Endosperm Development Are Affected by Heat Stress

**FIGURE 3:**
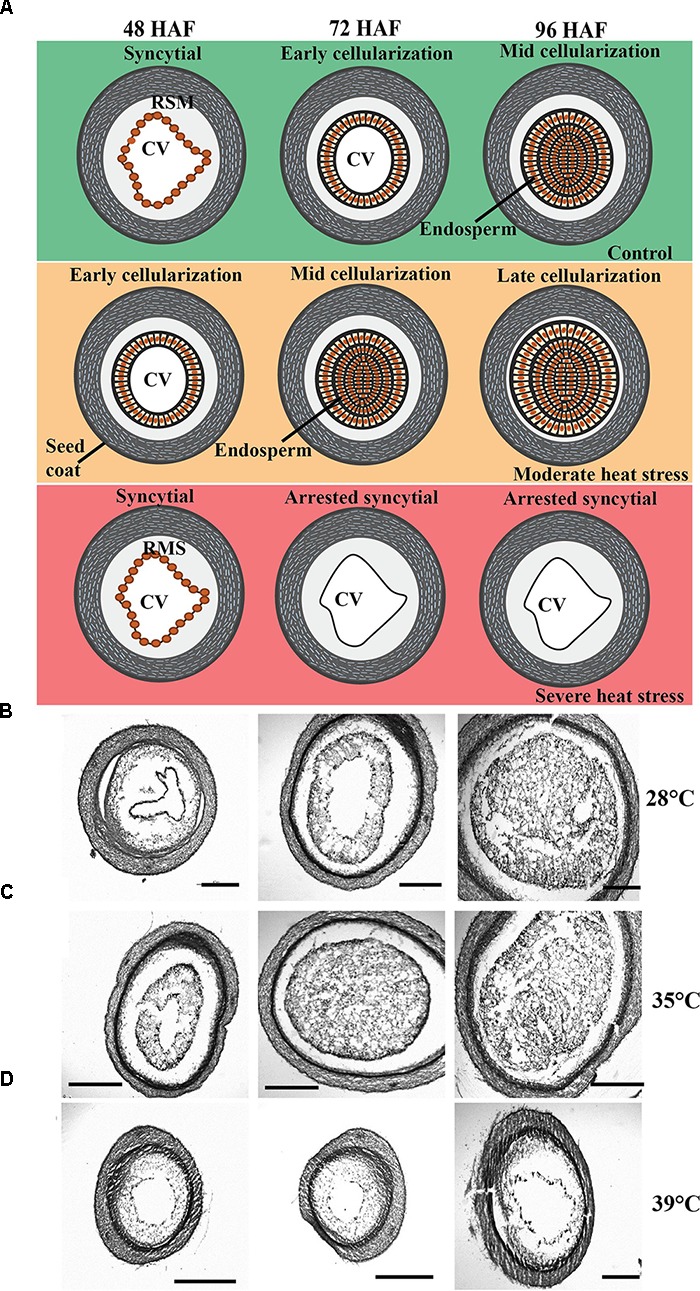
Effect of heat stress on developing rice seeds. **(A)** Schematic drawing showing early stages of endosperm (ED) development under non-, moderate, and severe HS conditions. **(B–D)** Histological analysis of early developing rice seeds under control conditions (28°C), moderate (35°C), and severe HS (39°C). Seeds were harvested at 48, 72, and 96 h after fertilization (HAF) for control, moderate, and severe HS. Upper row: sections of NS seeds **(B)**. Middle row: sections of moderate-stressed seeds **(C)** and bottom row: section of severe-stressed seeds **(D)**. Developing seeds at 48 HAF (Left), 72 HAF (Middle), and 96 HAF (Right). Moderate HS accelerated cellularization. Severe HS delayed cellularization. Radical Microtubule System (RMS), central vacuole (CV), ED. Scale bar = 200 μm.

Early seed development entails a transition from a syncytial ED, where nuclei are freely dividing without cytokinesis to initiation of cellularization. We collected developing seeds of control (28°C), moderate (35°C), and severe (39°C) HS plants and performed cross-sections (Figure 3). A schematic overview illustrating early seed development formation under control, moderate and severe HS is presented in Figure 3A. Under control conditions the overall pattern of rice seed development indicated syncytial ED stage at 48 HAF, followed by early cellularization stage at 72 HAF and mid cellularization by 96 HAF (Figure 3B). Under moderate HS, developing rice seeds exhibited accelerated cellularization and were completely cellularized at 72 HAF (Figure 3C). In contrast, seeds exposed to severe HS (39°C) failed to initiate cellularization 72 HAF and the central vacuole (CV) was still present at 96 HAF (Figure 3D). Our data suggest that 35°C accelerated and 39°C delayed the duration of the syncytial stage and transition toward cellularization, respectively.

### Transient Heat-Stress Alters the Seed Maturation Rate

To determine the impact of a transitory HS during early seed development on seed maturation, we measured the speed of ripening in control (28°C) and HS spikelets at 35 and 39°C. NS spikelets started to mature 25 days after fertilization (DAF), reaching 50% of maturation 31 DAF and 100% at 38 DAF. HS plants had faster maturation rates when compared with NS plants. Spikelets of moderate stressed plants for 24 h started to mature at 21 DAF, reaching more than 60% maturity at 27 DAF, and complete maturation by 31 DAF. Seeds that were HS for 48 h showed 45% maturation at 27 DAF and 100% maturation by 31 DAF (Supplementary Figure S1A). Seeds exposed to a severe HS for 72 h (Supplementary Figures S1B,C) did not reach ripening stage. Severe stress for 24 and 48 h resulted in 50 and 80% non-viable seeds. Therefore, both the intensity and duration of HS, however, transitory, impacted seed maturity.

### Short-Term Heat Stress During Development Alters Germination Rate

**FIGURE 4:**
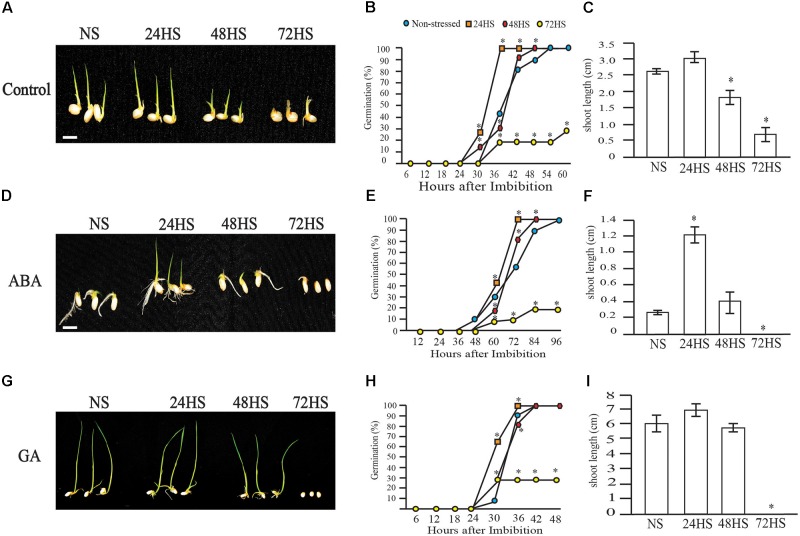
Altered hormone sensitivity of mature HS seeds during early seed development. **(A–C)** Moderate (35°C) HS seeds during early seed development display altered pattern of seed germination. **(A)** Five days after imbibition (DAI) of NS and HS rice seeds submitted to 24, 28, and 72 h of HS during early seed development. **(B)** Germination time course of NS and HS seeds. Germination rate was measured every 6 h starting after imbibition (HAI). **(C)** Shoot length of NS and HS rice seeds under control conditions measured at 5 DAI. Twenty-four hours of moderate HS (35°C) during early seed development reduces ABA sensitivity. **(D–F)** Germination analysis of control and stress seeds treated with 5 μM ABA. **(D)** Photographs show representative seedlings at 5 DAI, **(E)** germination rate, and **(F)** Shoot length at 5 DAI. Gibberellin (GA) did not influence seed germination after moderate HS imposition during early seed development. **(G–I)** germination analysis of seeds treated with 1 μM GA. **(G)** Photographs show representative seedlings at 8 DAI. **(H)** Germination rate and **(I)** Shoot length of GA treated seeds at 5 DAI. Representative graphs are shown (^∗^*p*-value < 0.0001, *n* = 20 seeds in each experiment). Bar = 1 cm.

Seed exposed to 39°C during early seed development were non-viable and failed to germinate (Figure 2A). Seeds exposed to 35°C during early seed development had different germination rates during a time course study (Figures 4A–C). Control seeds reached 42% germination 36 HAI and 100% at 54 HAI (Figure 4B). Seeds exposed to 24 h of HS during early seed development exhibited accelerated germination rate, reaching 30% at 30 HAI and 100% at 36 HAI (Figure 4B). Forty-eight hours of HS during early seed development resulted in a germination rate similar to NS seeds; 100% germination at 48 HAI (Figure 4B). A longer period of HS (72 h) was detrimental as only 30% of seeds germinated (Figure 4B).

### Effects of Exogenous ABA on Moderate Heat-Stressed Seeds

We next examined the ABA sensitivity of the seedlings obtained from control and HS seeds to determine if ABA responses are altered due to HS treatments. ABA delayed germination in control and HS seeds (Figures 4D,E). However, 24 h HS seeds were ABA-hyposensitive. The germination rate was higher in 24 h HS compared to control seeds, showing 100% germination at 72 HAI in 24 h HS seeds, while NS seeds reached 60% germination at the same time point. Seeds stressed for 48 h also had higher germination rate and reached 100% germination at 86 HAI. Seventy-two-hour HS seeds reached only 30% germination when treated with ABA (Figure 4E). These post-germination assay results indicate that 24 and 48 h HS seedlings were less sensitive to exogenous ABA. After 5 days of imbibition, shoot length was higher for seeds stressed for 24 h (Figure 4F), while control and 48 h HS seeds were clearly shorter (Figures 4D,F). After 8 days of ABA imbibition, 24 h HS seeds derived plants were taller compared with NS seedlings (Supplementary Figure S2).

### Effects of Exogenous GA on Moderate Heat-Stressed Seeds

Next, we examined the impact of HS on GA sensitivity during germination (Figures 4G–I). Control, 24 and 48 h HS seeds reached 100% germination at the end of the time course experiment. However, 72 h HS seeds only reached 30% germination (Figure 4H). At 30 HAI, 60% of 24 h HS seeds and 10% of NS seeds had geminated. The germination percentage of 48 and 72 h HS seeds was 28%. Post-germination assays showed no significant differences in shoot length among NS, 24 and 48 h HS seeds (Figures 4H,I), while 72 h HS seeds did not produce viable plants (Figure 4G). Collectively, this data suggests that GA affects the germination rate of HS and control seeds similarly.

### Transcript Level GA and ABA Crosstalk During Heat Stress

**FIGURE 5:**
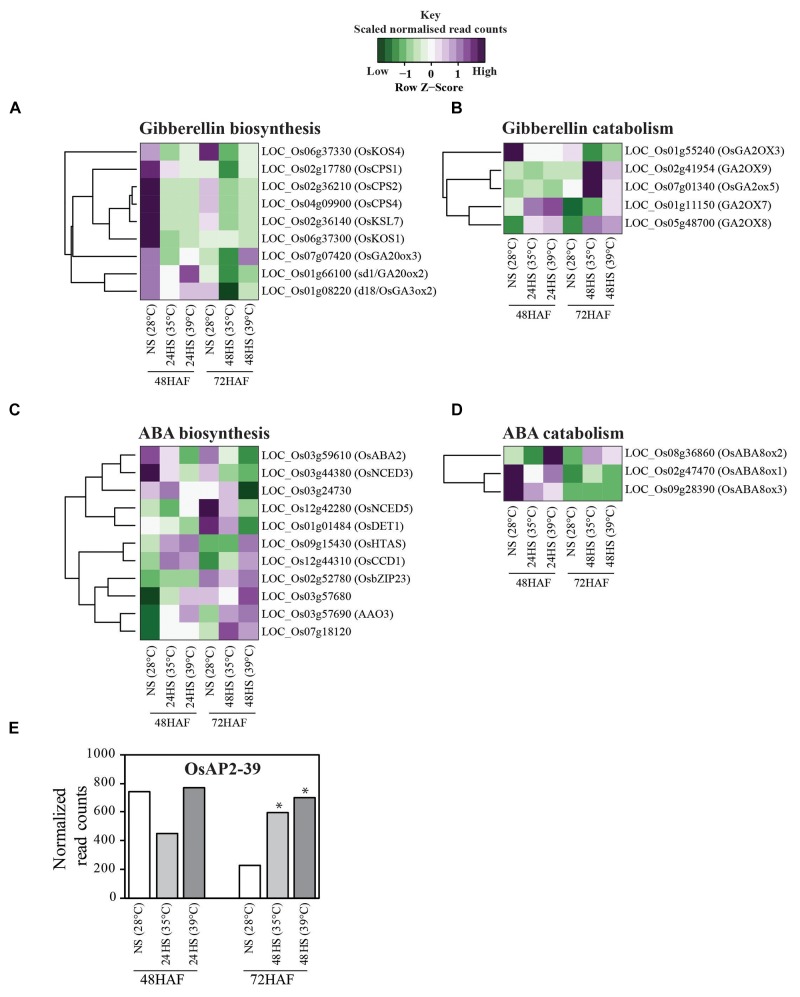
Heat stress induces transcriptional changes in ABA and GA related genes **(A–D)**. Heat maps displaying the thermal sensitivity of ABA and GA pathway genes involved in **(A)** GA biosynthesis, **(B)** GA catabolism, **(C)** ABA biosynthesis, and **(D)** ABA catabolism. **(E)** Bar graph represents normalized read counts of OsAP2-39 in seeds control and HS seeds. ^∗^Represents false discovery rate (FDR) < 0.0001.

Given the differential hormonal response of the seeds during germination, we queried the previously published RNAseq dataset for developing seeds growing under 35, 39°C, and control conditions sampled after 24 and 48 h of HS (Chen et al., 2016). We aimed to determine the level of transcriptional perturbation of GA and ABA pathway genes during the transient HS. The GA and ABA pathway genes that are differentially regulated between control and HS samples are listed in Supplementary Table S1. The gene annotation information for these two hormonal pathways was obtained from GoMapMan (Ramšak et al., 2014). Differentially expressed GA and ABA synthesis and catabolic pathway associated genes are represented as a heat map in Figures 5A–D. Moderate HS decreased the expression of GA biosynthesis genes (*GA20ox2* and *GA3ox2*) and increased the expression of genes encoding for GA catabolic enzymes (*GA2ox7* and *GA2ox8*). Expression of two ABA catabolic genes was higher in control and stressed samples at 48 HAF but was suppressed by 72 HAF. However, 24 h of moderate HS down-regulated another ABA catabolic gene, *OsABA8ox2* (*LOC_Os08g36860*) (Figure 5D). In context of GA and ABA crosstalk, we found the response of OsAP2-39 to moderate HS to be noteworthy. OsAP2-39 is a APETALA2 (AP2) class family transcription factor known for its role in regulating the GA and ABA antagonistic crosstalk in rice. OsAP2-39 controls the ABA-GA homeostasis by positively regulating the key enzymes OsNCED-1 and EUI, involved in ABA biosynthesis and GA deactivation, respectively (Yaish et al., 2010). We found that expression of OsAP2-39 in developing seeds to be induced by more than two-fold in 48 h moderate stressed seeds relative to corresponding control seeds (Figure 5E). At least at the gene expression level, the transcript abundance of *OsAP2-39* is consistent with overall increase in ABA biosynthesis and decreased GA biosynthesis. It is pertinent to note that change in expression of *OsAP2-39* in transgenic rice also affects the seed size (Yaish et al., 2010). Consistent with earlier reports, our data provides evidence that transient HS perturbs the antagonistic regulation of ABA and GA biosynthesis genes in young rice seeds.

### Hormone Content in Non- and Heat-Stressed Seeds

**FIGURE 6:**
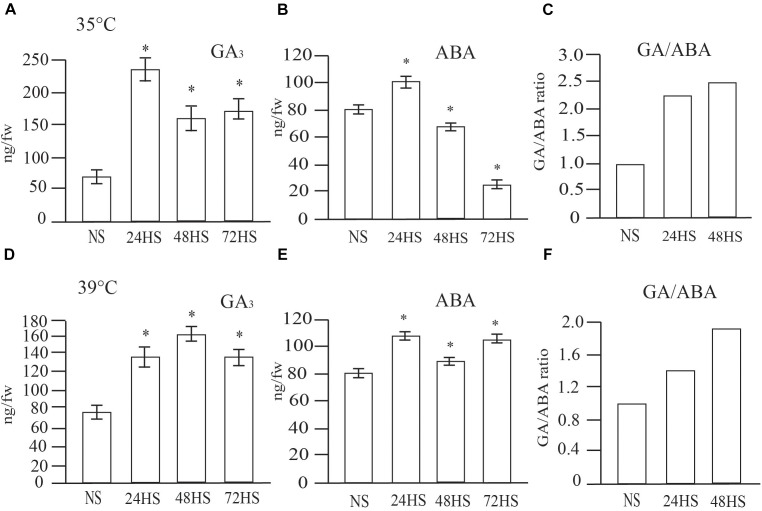
GA_3_ and ABA are altered in mature seeds when exposed to short-term periods of moderate and severe HS during early seed development. Seeds under **(A–C)** moderate and **(D,E)** severe HS. **(A)** GA_3_ levels in moderate HS seeds. **(B)** ABA levels in moderate HS seeds. **(C)** GA/ABA ratio of moderate HS seeds. **(D)** GA_3_ levels in severe HS seeds. **(E)** ABA levels in severe HS seeds. **(F)** GA/ABA ratio of severe HS seeds. Three independent experiments were carried out with similar result. Representative graphs are presented. Results are presented as means (*n* = 20 seedlings per experiment, ^∗^*p*-value < 0.0001).

Given the transcriptional perturbation of GA and ABA pathways genes during the transient HS, we next directly measured the hormone levels in control and HS seeds at maturity. We found significantly higher levels of GA_3_ in all seeds stressed at 35°C compared with NS seeds (Figure 6A). However, ABA levels in the same samples were reduced, except for the 24 h HS seeds (Figure 6B). Under severe HS (39°C), ABA and GA levels were significantly higher in all time points (Figures 6D,E). GA/ABA ratio was clearly altered under moderate and severe HS (Figures 6C–F), showing increase at 24 and 48 h of HS. However, 48 h of HS seeds had much higher GA/ABA ratio than seeds exposed to 24 h HS. These results suggest that while short term stress clearly alters the GA and ABA pathway genes and likely hormone levels, they are as expected, not the only developmental stage determining the final GA and ABA levels in seeds at maturity.

### Starch Content Is Altered in Heat-Stressed Seeds

**FIGURE 7:**
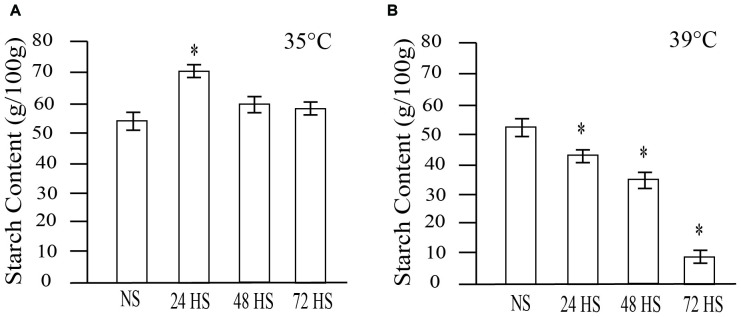
Effect of moderate (35°C) and severe (39°C) HS on total starch content in mature rice grains. **(A)** Total starch content in NS and moderate HS seeds. **(B)** Total starch content in non- and severe HS seeds. Three independent experiments were carried out with similar results. Representative graphs are presented. Results are presented as means (*n* = 20 seedlings per experiment, ^∗^*p*-value < 0.0001).

Starch is the main carbon source during germination and seedling establishment. We examined the impact of HS during early seed development on starch accumulation at maturity. Total starch content of non- and HS rice seeds is shown in Figure 7. NS seeds showed an average of 54 mg of starch/100 mg of dried seeds. Seeds HS at 35°C for 24 h during early seed development showed 14% higher total starch content (Figure 7A). Elevated total starch content in 24 h HS seeds could at least partly explain the accelerated germination speed (Figure 4B). HS seeds for 48 and 72 h showed a slight, but non-significant increase in starch content (Figure 7A). At 39°C, we observed a drastic reduction in total starch content which can be expected to have negative effects on germination. Thus, 24 h of HS at 35°C resulted in higher starch content, accelerated cellularization (Figure 3) and germination rate (Figure 4B). In contrast, any period of HS at 39°C during early seed development was detrimental to seed formation and maturation.

**FIGURE 8:**
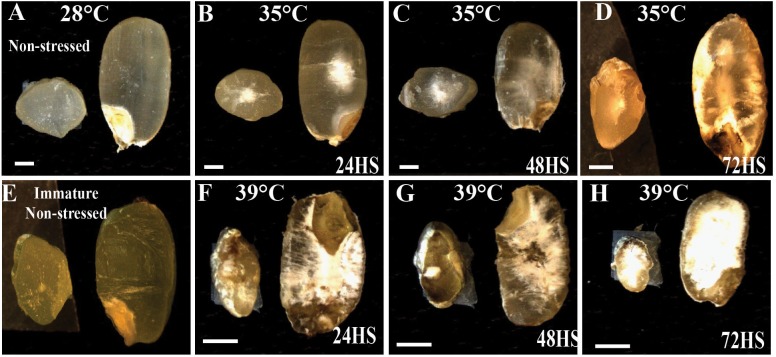
Effect of moderate (35°C) and severe (39°C) HS on rice grains. The longitudinal and cross sections are of mature rice seeds and represent (from left to right): **(A)** NS, **(B)** 24 HS at 35°C, **(C)** 48 HS at 35°C, **(D)** 72HS at 35°C, **(E)** NS immature seeds, **(F)** 24 HS at 39°C, **(G)** 48 HS at 39°C, and **(H)** 72 HS at 39°C. *n* = 20, Bar = 1 mm.

To further characterize the morphological changes prompted by the different levels of HS, we performed longitudinal and cross sections on mature seeds exposed to moderate and severe HS during early seed development (Figure 8). In NS seeds, a regular and uniform seed structure was observed (Figure 8A). The seeds showed variable signs of damage and increased severity of embryo damage at 72 HAF of 35°C HS (Figures 8B,C). We compared control and moderate HS seeds before maturation (Figure 8E). A starchy phenotype similar to mature seeds is also observed in immature seeds (Figures 8A,E). In contrast, under severe HS, we observed clear structural damage (Figures 8F–H). Our data indicate that moderate HS caused an increased accumulation of starch that likely promotes post-germination growth.

### Heat-Stressed Seeds Showed Altered Early Seedling Growth

**FIGURE 9:**
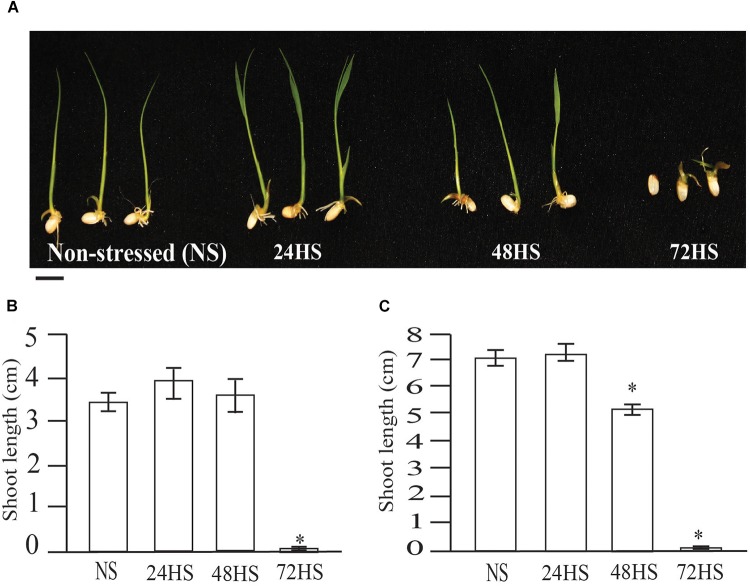
Moderate HS rice seeds during early seed development show altered phenotype during early seedling growth. **(A)** Shoot growth after 8 DAI. Photographs show representative seedlings. **(B)** Shoot length of control and HS seeds at 8 DAI. **(C)** Shoot length at 10 DAI. Five independent experiments were carried out with similar results. Representative graphs are presented. Results are presented as means (^∗^*p*-value < 0.0001, *n* = 20 seedlings per experiment). Bar = 1 cm.

To determine whether HS during early seed development affects seedling establishment, we measured seedling growth at eight and 10 DAI (Figure 9). Shoot length after 8 days of imbibition did not show significant differences among NS, 24 and 48 h HS seeds. In contrast, shoot length for seeds HS for 72 h were significantly shorter compared to NS seeds (Figure 9B). Interestingly, even though NS, 24 and 48 h HS plants did not display differences in shoot length, 24 h HS seedlings were developmentally advanced, due to difference in leaf number per plant (Figure 9A). At 10 days after imbibition, NS and 24 h HS seeds showed similar shoot length, however, 48 h HS seeds were significantly shorter (Figure 9C). Taken together, these data suggest that HS during early seed development alters the overall pattern of seed and plant development, speeding up during short periods of HS (24 h), moderately delayed at 48 h and severely detrimental after 72 h of HS during early seed development.

## Discussion

The sensitivity of ED development to environmental stresses has been shown in maize, rice, and wheat through transcriptome studies (Yu and Setter, 2003; Kakumanu et al., 2012; Folsom et al., 2014; Begcy and Walia, 2015; Chen et al., 2016). At the morphological level, our study showed high heat sensitivity of rice ED during the syncytial stage (Figures 1–4), which leads to precocious or delayed cellularization depending on stress severity. Understanding the effects of short periods of HS during early stages of seed development on mature seeds is necessary to improve tolerance and protect yield. Detrimental effects of HS were clearly observed on mature seeds (Figures 1, 2) and affected germination (Figure 4) and seedling establishment (Figure 9). Although an overall negative effect of HS on mature seeds was observed, seeds HS for 24 h displayed improved germination rate and seedling establishment in a manner similar to primed seeds.

A variety of seed priming methods including osmotic, chemical and plant hormones during pre-soaking, and other seed pretreatments have been shown to improve plant performance (Basra et al., 2006; Farooq et al., 2006; Hussain et al., 2017; Ruttanaruangboworn et al., 2017). For instance, GA_3_ and ethylene stimulated the elongation of embryonic tissues and internodes of rice seedlings while ABA promoted coleoptile elongation (Lee et al., 1998). Another well-established seed priming method is the application of a dry heat treatment on seeds to interrupt seed dormancy and ensure better seed germination (Dadlani and Seshu, 1990). In cotton and rice, short incubation periods of seeds under elevated temperatures (60°C) markedly improved seedling emergence and vigor (Basra et al., 2004, 2006). Our transient moderate HS treatment during early seed development improved seed germination rate and advanced seedling establishment, suggesting that short-term episodes of HS enhance seed performance. Both ABA and GA pathways are perturbed at gene expression level after 24 and 48 h of moderate HS (Figure 5). It is possible that the transient HS alters either the level or sensitivity of the seeds to ABA and/or GA, which persists in the next generation until hormone homeostasis is reestablished in response to developmental program and seedling’s environment. Several studies have indicated that germination-related genes are activated and maintained by priming treatments, indicating the role of pre-germination metabolic steps in germination (Gallardo et al., 2001; Soeda et al., 2005). Natural or artificial seed priming induces the mobilization and solubilizes a variety of compounds necessary for seed germination (Bailly et al., 1998; Capron et al., 2000; Gamboa-deBuen et al., 2006). Among other pre-germination metabolic changes, seed priming decreased the level of organic compounds (Bailly et al., 1998), changed fatty acids contents (Walters et al., 2005), and induced α-amylase production (Mwale et al., 2003). We observed an increased accumulation of total starch that may accelerate germination of 24 h HS seeds, resulting in enhanced emergence. ED storage, especially for starch is initiated once cellularization is complete (i.e., no CV persists). It is possible that precocious cellularization initiates early starch accumulation in 24 h HS seeds. However, early maturation of HS panicles observed in our experiments could potentially negate early cellularization enhancement for starch storage.

In cereals, the major storage compound is starch (Bewley, 1997; Shaik et al., 2014). Starch serves as the principal carbohydrate storage in most plants and can be rapidly mobilized to provide soluble sugars that can be used for germination and other metabolic processes (Bewley, 1997; Shaik et al., 2014). Abiotic stresses lead to a depletion in starch content and, consequently, a rise in the accumulation of soluble sugars (Krasensky and Jonak, 2012). We observed an altered germination speed and total starch accumulation (Figures 4, 7). Seeds germinated from plants exposed to moderate HS (35°C) for 24 h, showed higher germination rates (Figure 4B) which coincided with higher total starch content (Figure 7). Since most of the seed starch is remobilized to support germination (Bewley, 1997), this indicates a positive correlation between increased germination rate and the quantity of total starch available for the anabolic pathways required during germination and seedling establishment.

In non-climacteric fruits that have little or no ethylene requirement for ripening during fruit development, ABA has a stronger role; the decrease in auxin and the concomitant increase in ABA are significant events that signal the developmental transition to ripening (Jia et al., 2011). We observed an increase in ripening rate of 24 and 48 h HS spikelets suggesting a relation between accelerated speed in cellularization (Figure 3) and earlier ripening (Supplementary Figure S1A). Seeds that were HS for 24 h displayed higher ABA levels in mature seeds, which may indicate altered hormone balance on HS seeds. Our results show that 24 h of HS altered the GA/ABA ratio, which is consistent with our observation of increased germination speed (Figure 4B), and faster seedling establishment (Figure 9). Interestingly, 48 h of HS also displayed an altered ratio of GA/ABA, however, only in 24 h HS seeds an elevated starch concentration was observed. Inhibition of germination and maturation has been linked to an increase in up to two fold of the GA/ABA ratio in maize and Arabidopsis seeds (Koornneef et al., 1982; White et al., 2000). Additionally, ABA content in seeds was positively correlated with variations in dormancy in seeds with a similar degree of ABA sensitivity (Hilhorst, 1995; Bewley, 1997). Based on our results, we cannot clearly separate the impact of altered GA/ABA ratio and increased starch content on improved germination and seedling vigor of 24 h HS seeds.

During grain filling, ABA levels have been shown to influence the deposition of different seed compounds (Koornneef et al., 1982; Hilhorst, 1995; Bewley, 1997; Zhou et al., 2013). We observed an overall decrease in starch content when seeds were exposed to variable periods of moderate and severe HS during early seed development. However, 24 h of moderate HS increased total starch content (Figure 7), providing more resources for seed germination (Figures 4A,B) and seedling establishment (Figure 9). The duration of HS during seed development could have a big impact on subsequent generations. Studies focused on how HS affects the seed filling period suggest that the timing of stressful conditions might be decisive, since it could impact the biosynthesis of specific storage compounds (Yu et al., 2010). Interestingly, we found that HS on early developmental stages has a negative impact on storage compound accumulation. In wheat, it was demonstrated that drought stress during early seed development affects gene expression of storage compounds (Begcy and Walia, 2015), suggesting that different stresses during early seed development might modify the downstream expression of genes associated with the synthesis of seed storage compounds.

Predicted increase of global temperature in the future is expected to negatively impact food production in many regions. During reproductive development and particularly early seed development, exposure to stresses could limit plant productivity even more. Understanding the physiological and morphological responses of HS plants during early ED development integrated with molecular findings could help develop more resilient cultivars and agronomic practices.

## Author Contributions

KB and HW conceptualized the experiments. KB performed the experiments. JS and HW mined the transcriptome dataset. KB and HW wrote the paper. All authors read and approved the final manuscript.

## Conflict of Interest Statement

The authors declare that the research was conducted in the absence of any commercial or financial relationships that could be construed as a potential conflict of interest.

## References

[B1] Bailly C. Benamar A. Corbineau F. Come D. (1998). Free radical scavenging as affected by accelerated ageing and subsequent priming in sunflower seeds. *Physiol. Plant.* 104 646–652. 10.1034/j.1399-3054.1998.1040418.x

[B2] Basra S. M. A. Ashraf M. Iqbal N. Khaliq A. Ahmed R. (2004). Physiological and biochemical aspects of presowing heat stress on cottonseed. *Seed Sci. Technol.* 32 765–774. 10.15258/sst.2004.32.3.12

[B3] Basra S. M. A. Farooq M. Tabassum R. Ahmed N. (2006). Evaluation of seed vigour enhancement techniques on physiological and biochemical basis in coarse rice (*Oryza sativa* L.). *Seed Sci. Technol.* 34 719–728. 10.15258/sst.2006.34.3.18

[B4] Begcy K. Dresselhaus T. (2017). Tracking maize pollen development by the leaf collar method. *Plant Reprod.* 30 171–178. 10.1007/s00497-017-0311-4 29101473 PMC5701949

[B5] Begcy K. Dresselhaus T. (2018). Epigenetic responses to abiotic stresses during reproductive development in cereals. *Plant Reprod.* 31 343–355. 10.1007/s00497-018-0343-4 29943158 PMC6244825

[B6] Begcy K. Walia H. (2015). Drought stress delays endosperm development and misregulates genes associated with cytoskeleton organization and grain quality proteins in developing wheat seeds. *Plant Sci.* 240 109–119. 10.1016/j.plantsci.2015.08.024 26475192

[B7] Bewley J. D. (1997). Seed germination and dormancy. *Plant Cell* 9 1055–1066. 10.1105/tpc.9.7.1055 12237375 PMC156979

[B8] Borisjuk L. Rolletschek H. Radchuk R. Weschke W. Wobus U. Weber H. (2004). Seed development and differentiation: a role for metabolic regulation. *Plant Biol.* 6 375–386. 10.1055/s-2004-817908 15248120

[B9] Brunel-Muguet S. D’hooghe P. Bataille M. P. Larre C. Kim T. H. Trouverie J. (2015). Heat stress during seed filling interferes with sulfur restriction on grain composition and seed germination in oilseed rape (*Brassica napus* L.). *Front. Plant Sci.* 6:213. 10.3389/fpls.2015.00213 25914702 PMC4392296

[B10] Capron I. Corbineau F. Dacher F. Job C. Come D. Job D. (2000). Sugarbeet seed priming: effects of priming conditions on germination, solubilization of 11-S globulin and accumulation of LEA proteins. *Seed Sci. Res.* 10 243–254. 10.1017/S0960258500000271

[B11] Chen C. Begcy K. Liu K. Folsom J. J. Wang Z. Zhang C. (2016). Heat stress yields a unique MADS box transcription factor in determining seed size and thermal sensitivity. *Plant Physiol.* 171 606–622. 10.1104/pp.15.01992 26936896 PMC4854699

[B12] Dadlani M. Seshu D. V. (1990). Effect of wet and dry heat treatment on rice seeds germination and seedling vigor. *Int. Rice Res. Newslett.* 15 21–22.

[B13] Fahad S. Bajwa A. A. Nazir U. Anjum S. A. Farooq A. Zohaib A. (2017). Crop production under drought and heat stress: plant responses and management options. *Front. Plant Sci.* 8:1147. 10.3389/fpls.2017.01147 28706531 PMC5489704

[B14] Farooq M. Basra S. M. A. Tabassum R. Afzal I. (2006). Enhancing the performance of direct seeded fine rice by seed priming. *Plant Prod. Sci.* 9 446–456. 10.1626/pps.9.446

[B15] Finkelstein R. Reeves W. Ariizumi T. Steber C. (2008). Molecular aspects of seed dormancy. *Annu. Rev. Plant Biol.* 59 387–415. 10.1146/annurev.arplant.59.032607.092740 18257711

[B16] Folsom J. J. Begcy K. Hao X. Wang D. Walia H. (2014). Rice fertilization-independent endosperm1 regulates seed size under heat stress by controlling early endosperm development. *Plant Physiol.* 165 238–248. 10.1104/pp.113.232413 24590858 PMC4012583

[B17] Gallardo K. Job C. Groot S. P. C. Puype M. Demol H. Vandekerckhove J. (2001). Proteomic analysis of arabidopsis seed germination and priming. *Plant Physiol.* 126 835–848. 10.1104/pp.126.2.835 11402211 PMC111173

[B18] Gamboa-deBuen A. Cruz-Ortega R. Martinez-Barajas E. Sanchez-Coronado M. E. Orozco-Segovia A. (2006). Natural priming as an important metabolic event in the life history of *Wigandia urens* (Hydrophyllaceae) seeds. *Physiol. Plant.* 128 520–530. 10.1111/j.1399-3054.2006.00783.x

[B19] Grass L. Burris J. S. (1995). Effect of heat stress during seed development and maturation on wheat (Triticum durum) seed quality.2. mitochondrial respiration and nucleotide pools during early germination. *Can. J. Plant Sci.* 75 831–839. 10.4141/cjps95-139

[B20] Hilhorst H. W. M. (1995). A critical update on seed dormancy.1. primary dormancy. *Seed Sci. Res.* 5 61–73. 10.1017/S0960258500002634

[B21] Hofmann N. R. (2009). The plasma membrane as first responder to heat stress. *Plant Cell* 21:2544. 10.1105/tpc.109.210912 19773384 PMC2768915

[B22] Howell K. A. Narsai R. Carroll A. Ivanova A. Lohse M. Usadel B. (2009). Mapping metabolic and transcript temporal switches during germination in rice highlights specific transcription factors and the role of RNA instability in the germination process. *Plant Physiol.* 149 961–980. 10.1104/pp.108.129874 19074628 PMC2633829

[B23] Hu J. J. Rampitsch C. Bykova N. V. (2015). Advances in plant proteomics toward improvement of crop productivity and stress resistance. *Front. Plant Sci.* 6:209. 10.3389/fpls.2015.00209 25926838 PMC4396383

[B24] Hussain M. Farooq M. Lee D. J. (2017). Evaluating the role of seed priming in improving drought tolerance of pigmented and non-pigmented rice. *J. Agron. Crop Sci.* 203 269–276. 10.1111/jac.12195

[B25] Jia H. F. Chai Y. M. Li C. L. Lu D. Luo J. J. Qin L. (2011). Abscisic acid plays an important role in the regulation of strawberry fruit ripening. *Plant Physiol.* 157 188–199. 10.1104/pp.111.177311 21734113 PMC3165869

[B26] Kakumanu A. Ambavaram M. M. Klumas C. Krishnan A. Batlang U. Myers E. (2012). Effects of drought on gene expression in maize reproductive and leaf meristem tissue revealed by RNA-Seq. *Plant Physiol.* 160 846–867. 10.1104/pp.112.200444 22837360 PMC3461560

[B27] Khan R. A. (1976). Effect of high-temperature stress on the growth and seed characteristics of barley and cotton. *Basic Life Sci.* 8 319–324. 1032107 10.1007/978-1-4684-2886-5_29

[B28] Koornneef M. Jorna M. L. Derswan D. L. C. B. Karssen C. M. (1982). The isolation of abscisic-acid (Aba) deficient mutants by selection of induced revertants in non-germinating gibberellin sensitive lines of *Arabidopsis-Thaliana* (L) heynh. *Theor. Appl. Genet.* 61 385–393. 10.1007/BF00272861 24270501

[B29] Krasensky J. Jonak C. (2012). Drought, salt, and temperature stress-induced metabolic rearrangements and regulatory networks. *J. Exp. Bot.* 63 1593–1608. 10.1093/jxb/err460 22291134 PMC4359903

[B30] Lee S. S. Kim J. H. Hong S. B. Yun S. H. Park E. H. (1998). Priming effect of rice seeds on seedling establishment under adverse soil conditions. *Korean J. Crop Sci.* 43 194–198.

[B31] Li N. Xu R. Duan P. Li Y. (2018). Control of grain size in rice. *Plant Reprod.* 31 237–251. 10.1007/s00497-018-0333-6 29523952

[B32] Li T. Hasegawa T. Yin X. Y. Zhu Y. Boote K. Adam M. (2015). Uncertainties in predicting rice yield by current crop models under a wide range of climatic conditions. *Glob. Change Biol.* 21 1328–1341. 10.1111/gcb.12758 25294087

[B33] Liu Y. Fang J. Xu F. Chu J. Yan C. Schlappi M. R. (2014). Expression patterns of ABA and GA metabolism genes and hormone levels during rice seed development and imbibition: a comparison of dormant and non-dormant rice cultivars. *J. Genet. Genomics* 41 327–338. 10.1016/j.jgg.2014.04.004 24976122

[B34] Mwale S. S. Hamusimbi C. Mwansa K. (2003). Germination, emergence and growth of sunflower (Helianthus annuus L.) in response to osmotic seed priming. *Seed Sci. Technol.* 31 199–206. 10.15258/sst.2003.31.1.21

[B35] Rajjou L. Duval M. Gallardo K. Catusse J. Bally J. Job C. (2012). Seed germination and vigor. *Annu. Rev. Plant Biol.* 63 507–533. 10.1146/annurev-arplant-042811-105550 22136565

[B36] Ramšak Ž. Baebler Š. Rotter A. Korbar M. Mozetič I. Usadel B. (2014). GoMapMan: integration, consolidation and visualization of plant gene annotations within the MapMan ontology. *Nucleic Acids Res.* 42 D1167–D1175. 10.1093/nar/gkt1056 24194592 PMC3965006

[B37] Ruttanaruangboworn A. Chanprasert W. Tobunluepop P. Onwimol D. (2017). Effect of seed priming with different concentrations of potassium nitrate on the pattern of seed imbibition and germination of rice (*Oryza sativa* L.). *J. Integr. Agric.* 16 605–613. 10.1016/S2095-3119(16)61441-7

[B38] Saidi Y. Peter M. Finka A. Cicekli C. Vigh L. Goloubinoff P. (2010). Membrane lipid composition affects plant heat sensing and modulates Ca(2+)-dependent heat shock response. *Plant Signal. Behav.* 5 1530–1533. 10.4161/psb.5.12.13163 21139423 PMC3115095

[B39] Sangwan V. Orvar B. L. Beyerly J. Hirt H. Dhindsa R. S. (2002). Opposite changes in membrane fluidity mimic cold and heat stress activation of distinct plant MAP kinase pathways. *Plant J.* 31 629–638. 10.1046/j.1365-313X.2002.01384.x 12207652

[B40] Schmitz A. J. Begcy K. Sarath G. Walia H. (2015). Rice ovate family protein 2 (OFP2) alters hormonal homeostasis and vasculature development. *Plant Sci.* 241 177–188. 10.1016/j.plantsci.2015.10.011 26706069

[B41] Seck P. A. Diagne A. Mohanty S. Wopereis M. C. S. (2012). Crops that feed the world 7: rice. *Food Sec.* 4 7–24. 10.1007/s12571-012-0168-1

[B42] Shaik S. S. Carciofi M. Martens H. J. Hebelstrup K. H. Blennow A. (2014). Starch bioengineering affects cereal grain germination and seedling establishment. *J. Exp. Bot.* 65 2257–2270. 10.1093/jxb/eru107 24642850 PMC4036499

[B43] Soeda Y. Konings M. C. Vorst O. Van Houwelingen A. M. Stoopen G. M. Maliepaard C. A. (2005). Gene expression programs during *Brassica oleracea* seed maturation, osmopriming, and germination are indicators of progression of the germination process and the stress tolerance level. *Plant Physiol.* 137 354–368. 10.1104/pp.104.051664 15618428 PMC548865

[B44] Suzuki N. Miller G. Sejima H. Harper J. Mittler R. (2013). Enhanced seed production under prolonged heat stress conditions in Arabidopsis thaliana plants deficient in cytosolic ascorbate peroxidase 2. *J. Exp. Bot.* 64 253–263. 10.1093/jxb/ers335 23183257 PMC3528037

[B45] Tao F. Hayashi Y. Zhang Z. Sakamoto T. Yokozawa M. (2008). Global warming, rice production, and water use in China: developing a probabilistic assessment. *Agric. For. Meteorol.* 148 94–110. 10.1016/j.agrformet.2007.09.012

[B46] Toh S. Imamura A. Watanabe A. Nakabayashi K. Okamoto M. Jikumaru Y. (2008). High temperature-induced abscisic acid biosynthesis and its role in the inhibition of gibberellin action in Arabidopsis seeds. *Plant Physiol.* 146 1368–1385. 10.1104/pp.107.113738 18162586 PMC2259091

[B47] van Oort P. A. J. Zwart S. J. (2018). Impacts of climate change on rice production in Africa and causes of simulated yield changes. *Glob. Chang. Biol.* 24 1029–1045. 10.1111/gcb.13967 29230904 PMC5836867

[B48] Walters C. Landre P. Hill L. Corbineau F. Bailly C. (2005). Organization of lipid reserves in cotyledons of primed and aged sunflower seeds. *Planta* 222 397–407. 10.1007/s00425-005-1541-5 16136327

[B49] Wassmann R. Jagadish S. V. K. Heuer S. Ismail A. Redona E. Serraj R. (2009). Climate change affecting rice production: the physiological and agronomic basis for possible adaptation strategies. *Adv. Agron.* 101 59–122. 10.1016/S0065-2113(08)00802-X

[B50] White C. N. Proebsting W. M. Hedden P. Rivin C. J. (2000). Gibberellins and seed development in maize. I. Evidence that gibberellin/abscisic acid balance governs germination versus maturation pathways. *Plant Physiol.* 122 1081–1088. 10.1104/pp.122.4.1081 10759503 PMC58942

[B51] Whittle C. A. Otto S. P. Johnston M. O. Krochko J. E. (2009). Adaptive epigenetic memory of ancestral temperature regime in *Arabidopsis thaliana*. *Botany* 87 650–657. 10.1139/B09-030

[B52] Yaish M. W. El-kereamy A. Zhu T. Beatty P. H. Good A. G. Bi Y.-M. (2010). The APETALA-2-like transcription factor OsAP2-39 controls key interactions between abscisic acid and gibberellin in rice. *PLoS Genet.* 6:e1001098. 10.1371/journal.pgen.1001098 20838584 PMC2936520

[B53] Yang J. C. Zhang J. H. (2006). Grain filling of cereals under soil drying. *New Phytol.* 169 223–236. 10.1111/j.1469-8137.2005.01597.x 16411926

[B54] Yu B. Y. Gruber M. Khachatourians G. G. Hegedus D. D. Hannoufa A. (2010). Gene expression profiling of developing *Brassica napus* seed in relation to changes in major storage compounds. *Plant Sci.* 178 381–389. 10.1016/j.plantsci.2010.02.007

[B55] Yu L. X. Setter T. L. (2003). Comparative transcriptional profiling of placenta and endosperm in developing maize kernels in response to water deficit. *Plant Physiol.* 131 568–582. 10.1104/pp.014365 12586881 PMC166833

[B56] Zhou X. Yuan F. Wang M. Guo A. Zhang Y. Xie C. G. (2013). Molecular characterization of an ABA insensitive 5 orthologue in *Brassica oleracea*. *Biochem. Biophys. Res. Commun.* 430 1140–1146. 10.1016/j.bbrc.2012.12.023 23246838

