# Supplementary figures and images for: Transient Heat Stress During Early Seed Development Primes Germination and Seedling Establishment in Rice
Source: Front Plant Sci. 2018 Dec 5;9:1768. doi: 10.3389/fpls.2018.01768 (PMC6290647; doi:10.3389/fpls.2018.01768)

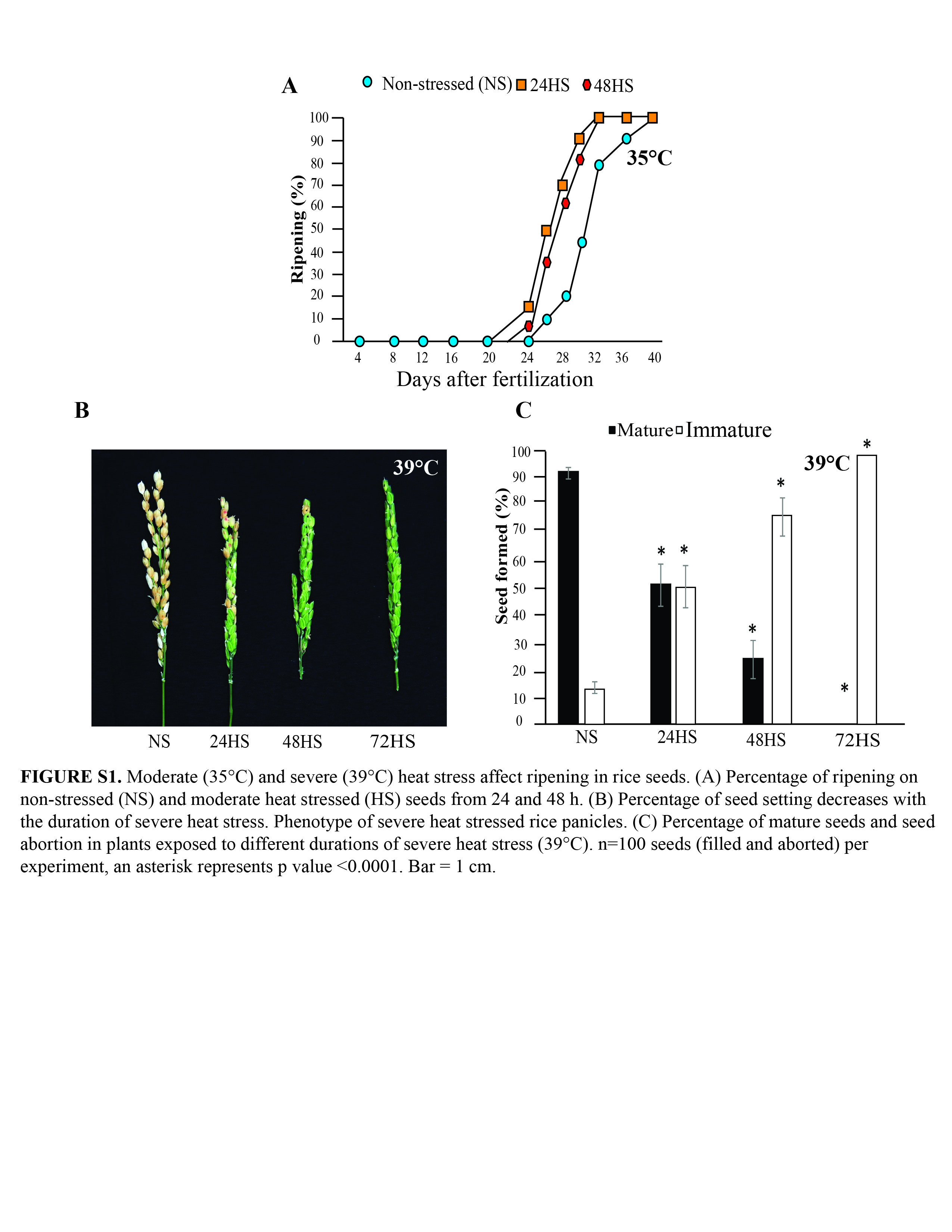

Supplement: Supplementary file 2 [file Image_1.JPEG]

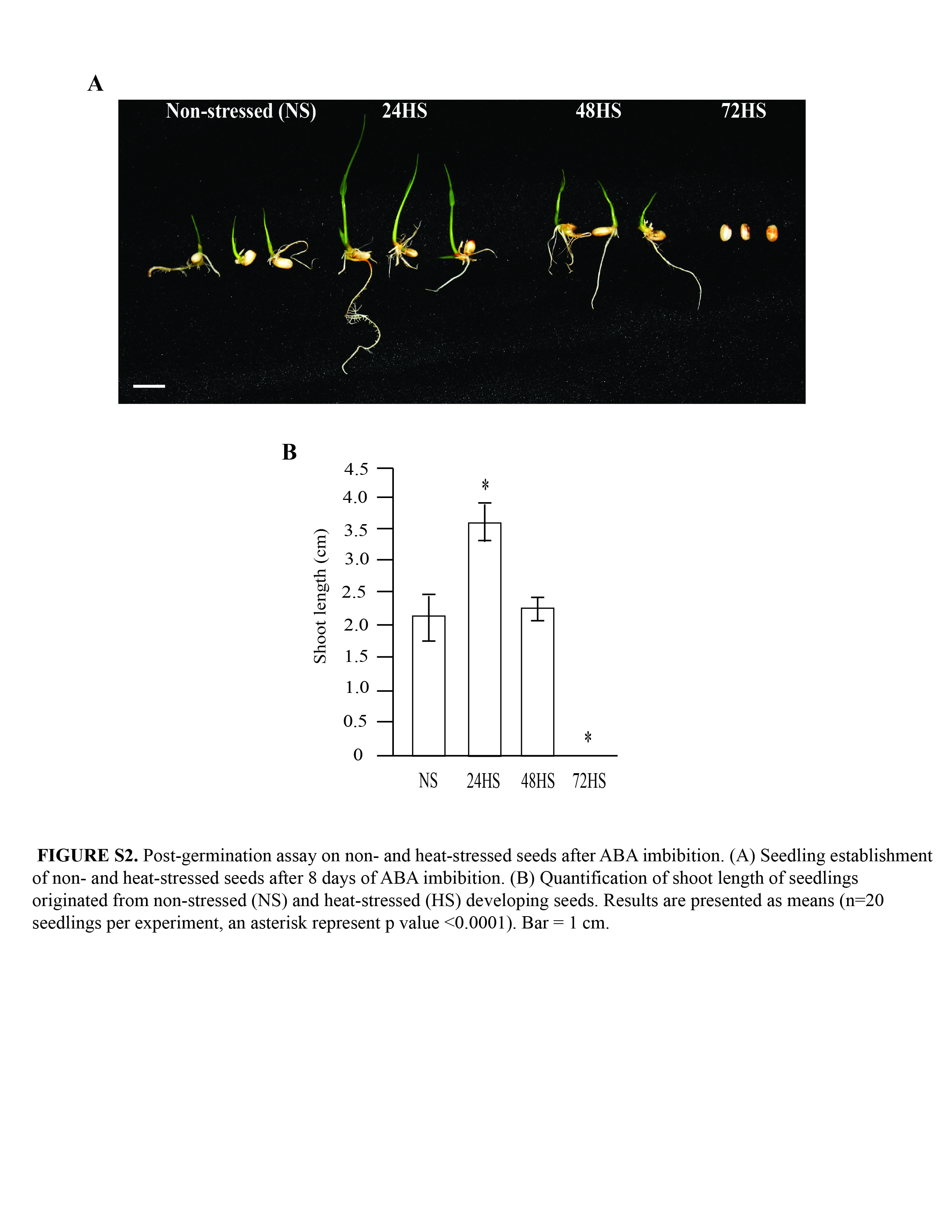

Supplement: Supplementary file 3 [file Image_2.JPEG]
